# Gender differences in innate responses and gene expression profiles in memory CD4 T cells are apparent very early during acute simian immunodeficiency virus infection

**DOI:** 10.1371/journal.pone.0221159

**Published:** 2019-09-06

**Authors:** Jeffy George, Ryan C. Johnson, Mary J. Mattapallil, Lynnsey Renn, Ronald Rabin, D. Scott Merrell, Joseph J. Mattapallil

**Affiliations:** 1 F. Edward Hébert School of Medicine, Uniformed Services University, Bethesda, Maryland, United States of America; 2 National Eye Institute, National Institutes of Health, Bethesda, Maryland, United States of America; 3 Center for Biologics Evaluation and Research, Food and Drug Administration, Silver Spring, Maryland, United States of America; CEA, FRANCE

## Abstract

Gender differences in Human immunodeficiency virus (HIV) disease progression and comorbidities have been extensively reported. Using the simian immunodeficiency virus (SIV) infected rhesus macaque model, we show that these differences are apparent very early during the course of infection. Though there were no major changes in the proportions of CD4 T cells or its subsets, central memory CD4 T cells from female macaques were found to differentially regulate a significantly larger number of genes at day 4 post-infection (PI) as compared to males. Pathway analysis revealed divergence of both canonical and biological pathways that persisted at day 10 PI. Changes in gene expression profiles were accompanied by a significant increase in plasma levels of pro-inflammatory mediators such as MCP-1/CCL2, I-TAC/CXCL11, and MIF. Though plasma levels of IFNα did not differ between male and female macaques, the expression levels of IFNα subtype-14, 16, IFNβ, and IFNω were significantly upregulated in the lymph nodes of female macaques at day 10 PI as compared to male macaques. Our results suggest that the pathogenic sequelae seen during chronic infection may be shaped by gender differences in immune responses induced very early during the course of HIV infection.

## Introduction

Human immunodeficiency virus (HIV) and simian immunodeficiency virus infection (SIV) is characterized by progressive loss of CD4 T cells leading to end stage AIDS[1–17]. The advent of HAART has had a significant impact on the course of HIV infection leading better long-term outcome for most patients. The effect of HIV infection, and impact of HAART over the course of disease has, however, not been uniform. This is especially the case with HIV infected women who have been reported to display higher levels of immune activation and progress to disease faster when compared to male subjects.

Numerous studies have shown that there were significant gender differences in the course of HIV pathogenesis[18–20] and plasma viral loads[21–23]. Interestingly, HIV infected female subjects display favorable clinical parameters during the initial stages of infection but later experience more adverse outcomes than their male counterparts[24].

T cells have been shown to differentially express gender biased genes at higher levels in women than men[25] with immune response genes such as IFNγ, Lymphotoxin-β, Granzyme A etc significantly over represented in women than men. Significant gender based differences in anti-viral cytokine responses induced by memory CD4 T cells have been reported[26]. Expression of interferon stimulated genes (ISG), IFNα, and immune activation relative to plasma HIV RNA loads were significantly higher in HIV infected female subjects as compared to male subjects[27–29]. The above studies suggest that gender differences are a potential determinant of pathogenic outcomes following HIV infection though it is not clear if these differences are apparent during the early stages of infection.

We sought to address this question using the rhesus macaque model for HIV infection. Rhesus macaques have been extensively used to study HIV pathogenesis[1–5, 7–10, 14, 30–49]. Our results show that central memory (CM) CD4 T cells from SIV infected female macaques differentially regulate a significantly larger number of genes at day 4 post-infection (PI) as compared to males. These differences were still apparent at day 10 PI, albeit with fewer number of genes. Changes in gene expression profiles were accompanied by significantly elevated levels of innate inflammatory cytokines such as MCP-1, I-TAC, and MIF in the plasma of female macaques. Though there was no difference in plasma levels of IFNα between males and females, levels of IFNα subtype-14, 16, IFNβ, and IFNω expression were significantly elevated at day 10 PI in the lymph nodes (LN) of female macaques as compared to male macaques.

## Materials and methods

### Animals, infection and samples

A total of 4 male and 4 female rhesus macaques (*Macaca mulatta*) of Indian origin seronegative for SIV, simian retrovirus (SRV) and simian T-cell leukemia virus (STLV) type-1 were used in this study. Animals were obtained from Bioqual, Inc. MD and housed at Bioqual in accordance with the recommendations of the Association for Assessment and Accreditation of Laboratory Animal Care International Standards and NIH Guide for the Care and Use of Laboratory Animals of the United States. The Institutional Animal Use and Care Committee of BIOQUAL approved these experiments (protocol #11-3528-01) followed by USUHS IACUC. When immobilization was necessary for procedures, the animals were sedated intramuscularly with 10 mg/kg of Ketamine HCl (Parke-Davis, Morris Plains N.J.) before any direct handling or procedures. All efforts were made to minimize suffering. Details of animal welfare and steps taken to ameliorate suffering were in accordance with the recommendations of the Weatherall report, “The use of non-human primates (NHP) in research”. Animals were housed in an air-conditioned facility with an ambient temperature of 21–25°C, a relative humidity of 40%–60% and a 12 h light/dark cycle. Animals were socially housed when possible or individually housed if no compatible pairing could be found. The animals were housed in suspended stainless steel wire-bottomed 6 sq ft cages and provided with a commercial primate diet and fresh fruit and vegetables twice daily with water freely available at all times. Social housing, toys, foraging equipment and mirrors were provided. Animals were monitored at least twice daily for behavior, food intake, activity, and overall health by trained technicians. When the animals were euthanized, they were first sedated with ketamine and then given an overdose of pentobarbital.

All animals were infected intravenously at the same time with ~100 animal infectious doses of SIVmac251 that was obtained from Dr. Norman Letvin at Harvard Medical School. Peripheral blood and plasma samples were collected longitudinally from each animal at day 0, 4 and 10 PI. All the animals were sacrificed at day 10 PI, and blood and tissue samples were collected for analysis.

PBMC was isolated from peripheral blood by density gradient centrifugation, whereas cells from LN were isolated by mechanical disruption. Plasma viral loads were determined by real-time PCR using reverse-transcribed viral RNA as the template, as previously described [50].

### Antibodies and flow cytometry

Isolated PBMC were labeled with a panel of CD3-Cy-7APC, CD4-APC, CD8-Alexa700, CD20-Pacific Blue, CD95-FITC and CD28-Cy-5PE (BD Biosciences) antibodies and analyzed using a BD LSR II instrument. Central memory CD4 T cells (CD3+CD20-CD4+CD28+CD95+) were sorted using a Becton Dickinson Aria sorter, and used for microarray analysis. Memory CD4 T cells was discriminated based on the expression of CD28 and CD95 as described previously[7, 51]. After excluding dead cells, live CM cell subsets that were sufficient to yield a minimum of ~1 ug of RNA were sorted (>95% purity) and used for RNA extraction.

### Microarray hybridization and analysis

Approximately 500 ng of purified RNA obtained from CM CD4 T cells at day 0, 4 and 10 PI was transferred to The Sidney Kimmel Cancer Center Microarray Core Facility at Johns Hopkins University (NIH grant P30 CA006973) for processing. RNA was assessed for quality using 2100 Bioanalyzer (Agilent Technologies, Santa Clara, CA), amplified and labeled using the Quick RNA Amplification and Labeling Kit (Agilent Technologies) with minor modifications. Briefly, 400 ng total RNA was reverse-transcribed into cDNA by MMLV-RT using oligo-dT primers (System Bioscience, Palo Alto, CA) that incorporate the T7 promoter sequence. The cDNA was *in vitro* transcribed in the presence of T7 RNA polymerase and Cyanine (Cy5 or Cy3)-labeled CTP (Perkin Elmer, Pittsburg, PA). The labeled cRNA was purified using the RNeasy mini kit (Qiagen). RNA spike-in controls (Agilent Technologies) were added to each RNA sample before amplification and labeling.

Samples were hybridized to the Agilent Rhesus macaque Gene Expression Microarrays, 4x44k, P/N G2519F, V1 (Agilent Microarray Design ID 015421) using each animal’s day 0 sample as the reference to normalize for the day 4 and 10 post-infection time points from that same animal. A total of 800 ng of each Cyanine-labeled sample was used for hybridization at 65°C for 17 hours in a rotating hybridization oven.

After hybridization, the microarray slides were washed, dried in an ozone-controlled enclosure, and scanned and analyzed using an Agilent G2505C Scanner controlled by Agilent Scan Control 7.0 software. After extraction with the Agilent Feature Extraction 9.5.3.1 software, data was stored initially in the Stanford Microarray Database[52], and subsequently moved to the Gene Expression Omnibus (GEO) and are available as accession GSE38917 at https://www.ncbi.nlm.nih.gov/geo/query/acc.cgi?acc=GSE38917.

For analysis, the log_2_ of red/ green normalized ratios for the day 4 and 10 samples (normalized to day 0 of each animal) for all non-flagged features were recovered by their Biosequence ID. The resulting data were averaged by sex (male and female) and day (day 4 and 10). Spots with a significant difference in average ratio (two-sided t-test, p < 0.05) between males and females at either day 4, or day 10 were extracted from the data set, and subjected to hierarchical cluster analysis. The clustering was performed and visualized using the superheat R package (superheat v0.1.0, https://github.com/rlbarter/superheat).

Significant changes in spot signal ratios between males and females were additionally visualized using volcano plots. Briefly, changes in mean log_2_ signal ratios were calculated at day 4 and day 10 and plotted along the x-axis. Two-sided t-tests were used to assess significant differences between males and females for each spot, and the negative log p-values were plotted along the y-axis. Horizontal dashed and solid black lines that denote p-values at 0.1 and 0.05, respectively, were added to the volcano plot. Red spots to the left of 0 (vertical dashed red line) were more upregulated in females than males, and blue spots to the right of 0 were more upregulated in males than females. Black points above the significance lines represent genes that were significantly more down regulated in males (right side) or females (left side). All analyses were performed using R software (version 3.5.1). Significantly differentially regulated genes in male and female macaques at day 4 and 10 PI were used to identify canonical, and molecular/ cellular function pathways using the Ingenuity Pathway Analysis software (Qiagen).

### Cytokine levels in plasma

Plasma cytokine levels were determined at the Immunology Unit of the Duke Human Vaccine Institute using the Cytokine Monkey Magnetic 29-Plex Panel for Luminex^™^ Platform (Thermofisher Scientific, Waltham, MA). This kit can simultaneously quantify 29 cytokines namely, FGF-basic, IL-1β, G-CSF, IL-10, IL-6, IL-12, RANTES, Eotaxin, IL-17, MIP-1α, GM-CSF, MIP-1β, MCP-1, IL-15, EGF, IL-5, HGF, VEGF, IFNγ, MDC, I-TAC, MIF, IL-1RA, TNFα, IL-2, IP-10, MIG, IL-4 and IL-8 in rhesus macaques[53, 54]. Plasma samples were setup in triplicates and the plates were analyzed using Luminex xMAP technology on a Bio-plex 200 system (Biorad).

Plasma levels of IFNα were determined using the human IFNα pan ELISA kit (Mabtech, Cincinnati, OH) with a limit of detection of 4 pg/ ml.

### Absolute quantification of *Macaca mulatta* IFN subtype mRNA levels by qRT-PCR

The absolute expression of Type I and, III IFN subtypes were determined as described previously[33, 34]. Briefly RNA isolated from LN cells using the RNeasy Mini Kit (Qiagen, Valencia, CA), DNase (Qiagen) was reverse transcribed (total RNA 500 ηg) with the Verso cDNA Synthesis Kit (Thermo Scientific, Rockford, IL) with a combination of random hexamers and anchored oligo dT primers at 42°C for 30 min, 95°C for 2 min, 4°C for ∞. RNase H (New England Biolabs, Ipswich, MA) was included in each sample.

The expression levels of type I and III IFN was determined by qRT-PCR using type I and III interferon subtype (IFNα-01/13, 02, 06, 08, 14, 16, 23, 24, 25, 26, 27, 28, 29, IFNβ, IFNω and IFNλ-1) primers and probes that were specific for *Macaca mulatta*[55]. Taqman Fast Universal PCR Master Mix and the primer/probe sets for housekeeping genes GAPDH and 18S obtained from Applied Biosystems (Foster City, CA) were used as controls. Four point standard curves of linearized plasmids containing the IFN subtype sequences as inserts and no template controls were included on each assay plate. The qRT-PCR assay plates were analyzed using a ViiA 7 Real-Time PCR System (Life Technologies, Grand Island, NY) at 50°C for 2 min, 95°C for 10 min, 40 cycles of 95°C for 15 sec, 60°C for 1 min, and collected data was analyzed using the ViiA 7 RUO Software (Life Technologies). The absolute numbers of each IFN transcript was calculated using standard curves and normalized to micrograms of RNA input per well.

### Data analysis

Flow cytometric data was analyzed using FlowJo version 9.2 (Tree Star, Inc., Ashland, OR). Statistical analysis was performed using Graph Pad Prism Version 5.0 software (Graph Pad Prism Software, Inc. San Diego, CA). Differences in CD4 T cell dynamics and cytokine profiles between time points were determined using One-way ANOVA followed by post-hoc analysis using Tukey’s multiple comparisons test. Differences in IFNα expression levels between male and female macaques were determined using *Mann-Whitney U test*. A *p < 0*.*05* was considered significant.

## Results

### Acute plasma viral loads and CD4 T cell subset dynamics do not significantly differ between male and female macaques

**Fig 1:**
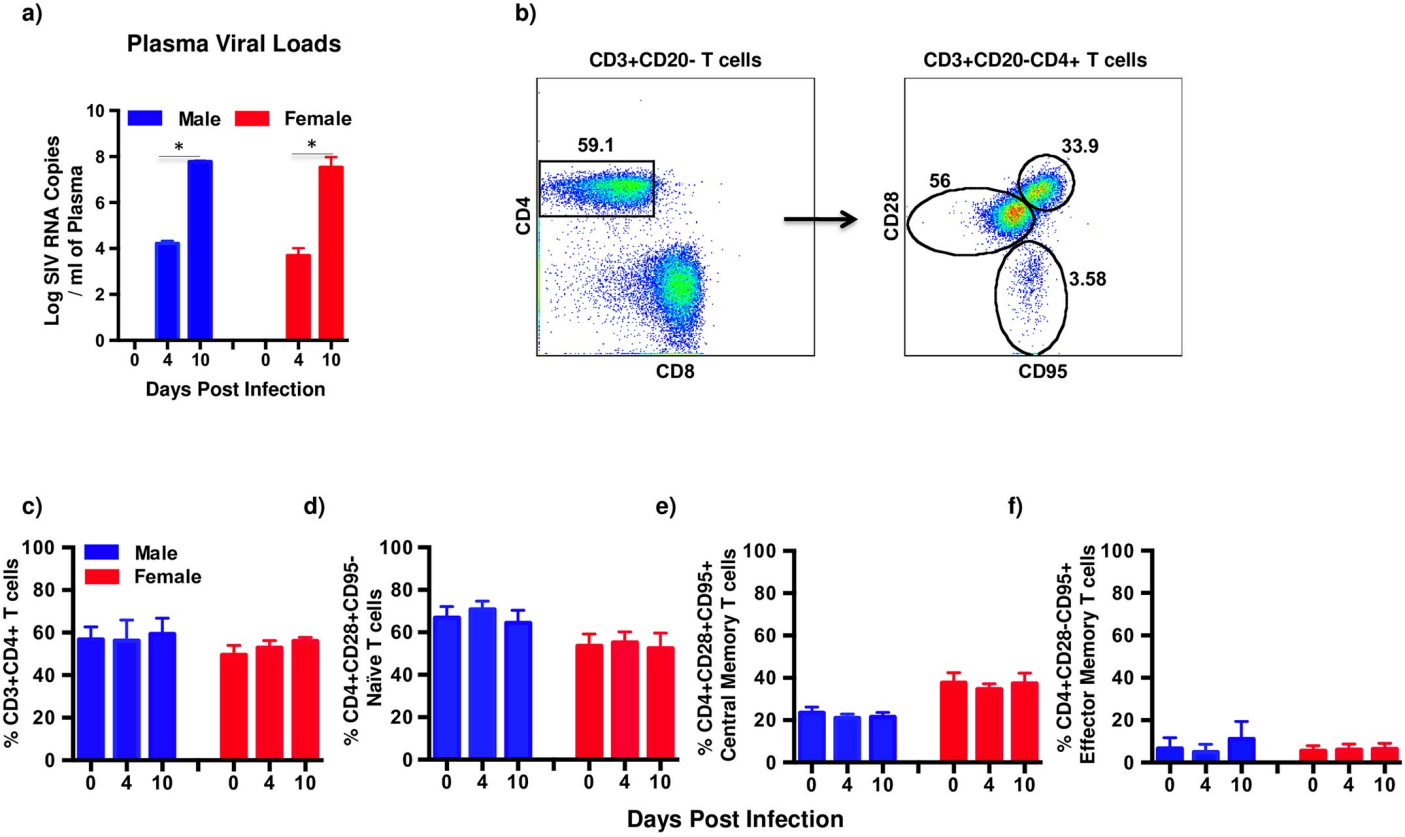
Plasma viral loads and CD4 T cell dynamics are similar in male and female macaques. (a) Plasma viral loads (Limit of detection is 30 copies /ml of plasma) in male (n = 4) and female (n = 4) macaques at day 4 and 10 post SIV infection (PI). (b) Representative dot plots showing the discrimination of CD4 T cells and it subsets in peripheral blood. Naïve and memory CD4 T cells were discriminated based on the differential expression of CD28 and CD95 on CD4 T cells. The proportion of peripheral blood (c) CD3+CD20-CD4 T cells, (d) CD4+CD28+CD95- naïve CD4 T cells, (e) CD4+CD28+CD95+ central memory CD4 T cells, and (f) CD4+CD28-CD95+ effector memory CD4 T cells at day 0, 4 and 10 PI in male (n = 4) and female (n = 4) macaques. Error bars represent standard error.

Previous studies[21–23] have reported that female HIV infected subjects have lower plasma HIV loads compared to male subjects. To determine if these differences were apparent during the acute stages of infection, we examined plasma viral loads in female macaques at day 4 and 10 PI and compared them to male macaques (Fig 1a). Plasma viral loads were readily detectable at day 4 PI and significantly increased by day 10 PI that did not significantly differ between the male and female groups of animals.

Next we examined the dynamics of CD4 T cell subsets to determine if acute SIV infection was associated with gender dependent changes in these subsets. Naïve (CD28+CD95-), central memory (CD28+CD95+), and effector memory (CD28-CD95+) CD4 T cells were discriminated based on the expression of CD28 and CD95 (Fig 1b) as described previously. There were no significant differences in the proportions of either total CD4 T cells or naïve and memory subsets following SIV infection between male and female macaques as compared to day 0 values (Fig 1c–1f).

### Significantly larger numbers of genes are differentially regulated in central memory CD4 T cells from female macaques as compared to males

**Fig 2:**
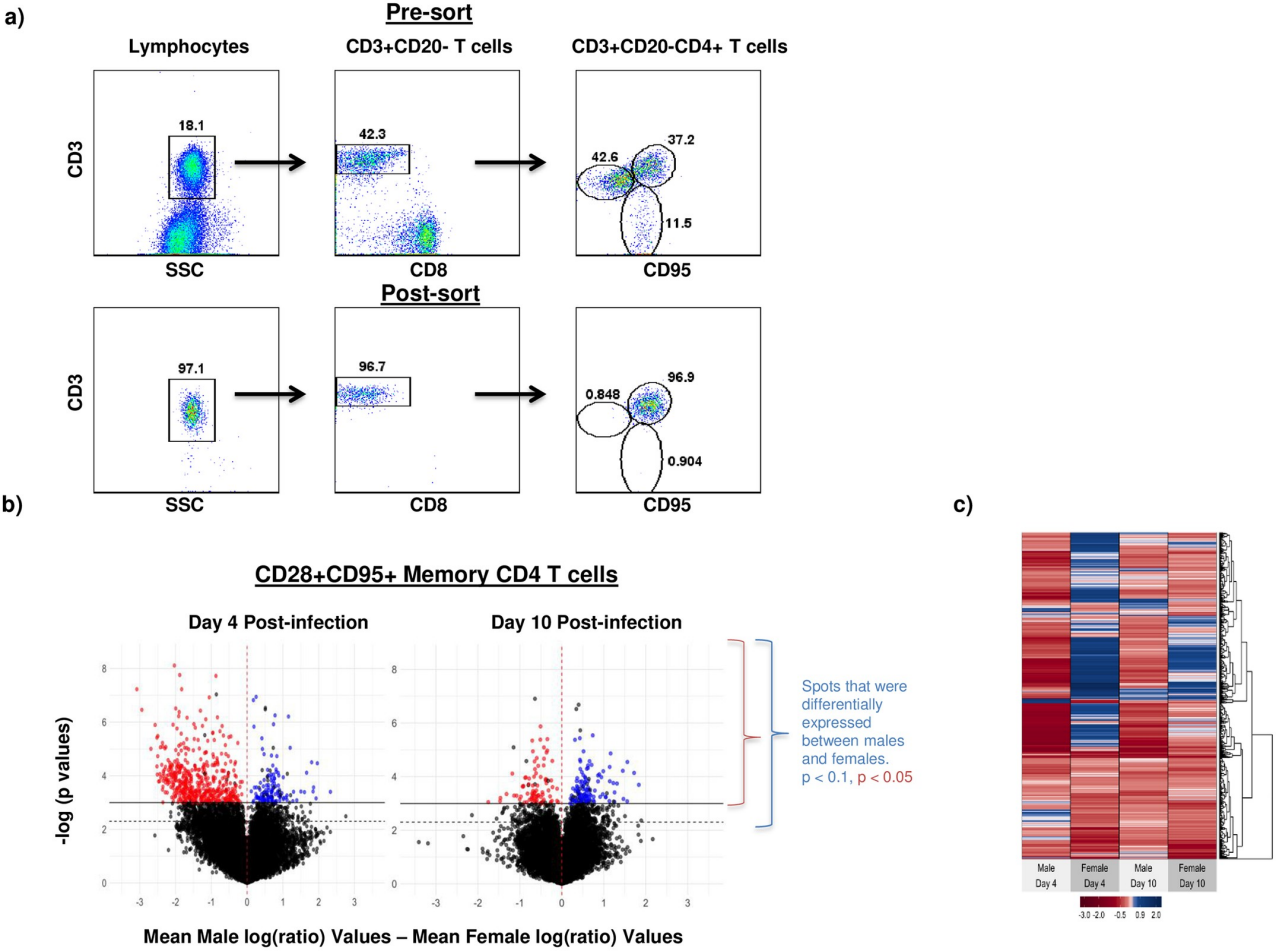
Female macaques differentially regulate significantly larger number of genes during acute SIV infection as compared to male macaques. (a) Representative dot plots showing the sorting strategy (Pre-sort) used to obtain populations of central memory (CM) CD4 T cells and the purity of the CM CD4 T cells (Post-sort). (b) Volcano plots and (c) heat map generated using genes that were differentially regulated in CM CD4 T cells from male (n = 4) and female (n = 4) macaques at day 4 and 10 post SIV infection. Each animal’s day 0 sorted CM CD4 T cell sample was used to normalize the same animals day 4 and 10 post SIV infection sorted samples. For (b), the mean log2 signal ratio (e.g. log2(Day 4 signal/ Day 0 signal)) for each gene on the microarray was calculated for each gender (4 animals per gender) and the difference in mean ratio values was computed between males and females and plotted along the x-axis. Genes that exhibited significant differences in expression between males and females are plotted above the horizontal significance line (solid line: p < 0.05; dashed line: p < 0.1). Red colored points indicate genes that were more upregulated in females than males, and blue points represent genes that were more upregulated in males than females. Black points falling above the significance lines indicate those points that are more down regulated between males and females. Black points above the significance line represent genes that were significantly more down regulated between males (left side) and females (right side).

Memory CD4 T cells is the primary target for both HIV and SIV infections. To determine if acute SIV infection was associated with changes in the profile of gene expression, we sorted highly purified populations of central memory (CM) CD4 T cells (Fig 2a) and used their RNA for microarray analysis. The mean log2 signal ratio (e.g. log2(Day 4 signal / Day 0 signal for each animal) for each gene on the microarray was calculated.

When considering how gene expression changed in the animals in relation to the day 0 data for the same animal, several trends were observed. For example, at day 4 PI, females exhibited a higher number of up-regulated genes (2-fold increase or more) as compared to males (69 versus 7 genes, respectively). By day 10 PI, this gender-specific difference in upregulated genes was retained, but the number of genes was diminished (22 genes in females versus 6 genes in males). In total, only 5 genes were similarly upregulated in males and females at day 4 and/or day 10 PI (S1 Table). As compared to the number of up-regulated genes, down-regulated genes (2-fold decrease or more) were more abundant in both genders at both time points PI. At day 4 PI, 125 male and 89 female downregulated genes were observed, and 73 male and 102 females down-regulated genes were detected at day 10 PI. We also observed significant overlap in down-regulated genes between the genders; 37 at day 4 PI, and 45 at day 10 PI. A table showing the gender-specific differences and similarities in up and down-regulated genes is provided (S1 Table).

The difference in mean ratio values between males and females were calculated and plotted along the x-axis in Fig 2b. Genes that exhibited significant differences in expression between males and females are plotted above the horizontal significance line (solid line: p < 0.05). In total, we observed 633 genes at day 4 PI that were significantly differentially expressed (p < 0.05) between males and females, and 216 genes at day 10 PI (S2 and S3 Tables). This increase in differential gene expression at day 4 PI was largely driven by an increased number of genes that were significantly more upregulated in females (484, red spots; Fig 2b) as compared to males (118, blue spots; Fig 2b).

Individual mean log2 ratio values for all genes that were differentially expressed between males and female at either day 4 or 10 PI (all points above the solid line (p < 0.05) in Fig 2b) was visualized in a heatmap (Fig 2c). The day 4 and 10 PI data were obtained after normalizing to each animal’s day 0 sample. At day 4 PI, a large proportion of genes were significantly upregulated in females as compared to males. Intriguingly, a large subset of these genes, were downregulated in males at day 4 PI. By day 10 PI, this altered change in gene expression diminished, but hierarchical clustering of genes revealed subsets of genes that retained their differential expression patterns between males and females. The expression profiles for the differentially expressed genes for each individual animal is provided in S2 and S3 Tables and can be visualized in S1 Fig.

**Fig 3:**
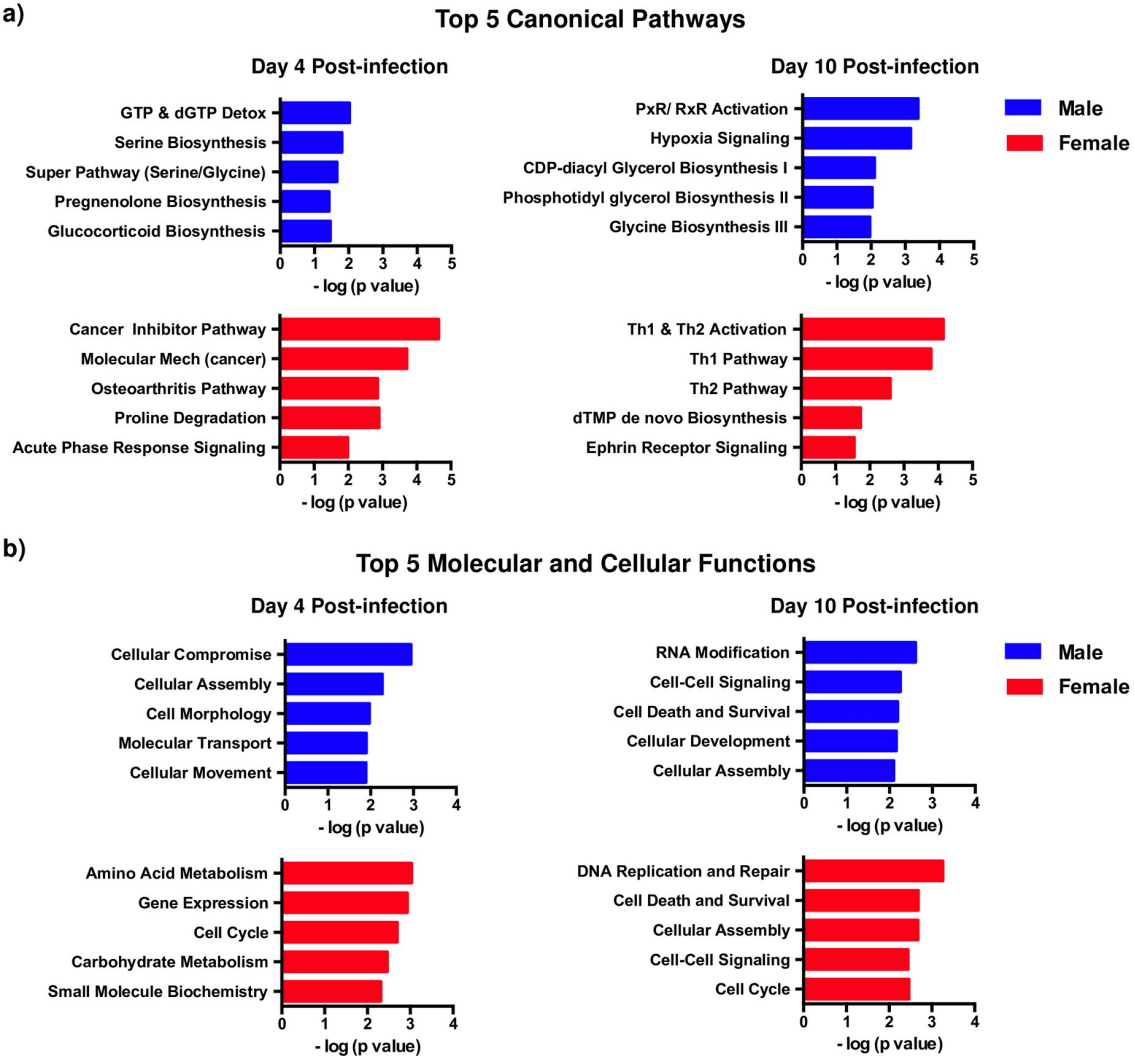
Canonical and biological pathways in memory CD4 T cells diverge in male and female macaques during acute SIV infection. Statistically significant genes that were differentially regulated between male and female macaques were used to pathway analysis using Ingenuity Pathway Analysis software. (a) Top 5 canonical pathways in (n = 4) and female (n = 4) macaques at day 4 and 10 post SIV infection (PI), and (b) top 5 molecular and cellular functions in (n = 4) and female (n = 4) macaques at day 4 and 10 PI. Each animal’s day 0 sorted CM CD4 T cell sample was used to normalize the same animals day 4 and 10 post SIV infection sorted samples.

To assess if differential regulation of genes was associated with differences in specific biological pathways and functions, we performed pathway analysis of differentially genes in female macaques at day 4 and 10 PI and compared them to male macaques (Fig 3a and 3b). Our results showed that the genes associated with both canonical pathways and molecular/ cellular functions diverged between male and female macaques suggesting that acute SIV infection differentially impacts gene expression in CD4 T cells from male and females during the very early stages of infection.

### Plasma levels of MCP-1, I-TAC, and MIF are significantly elevated in female macaques at day 10 PI as compared to male macaques

**Fig 4:**
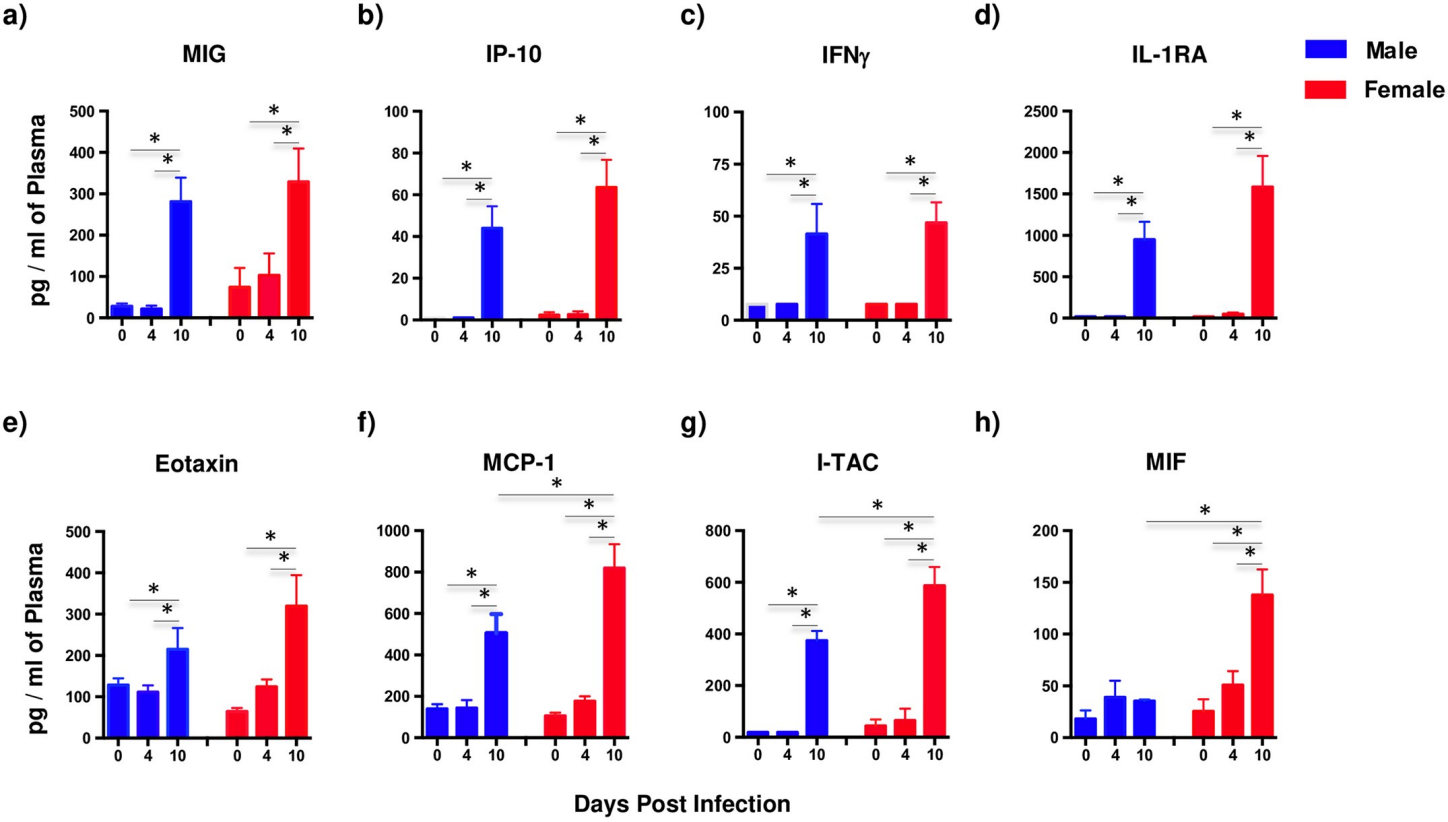
Plasma levels of MCP-1, I-TAC and MIF are significantly upregulated in female macaques as compared to male macaques during acute SIV infection. Levels of (a) MIG, (b) IP-10, (c) IFNγ, (d) IL-1RA, (e) Eotaxin, (f) MCP-1, (g) I-TAC, and (h) MIF in the plasma samples that were collected from each animal at day 0, 4 and 10 post-SIV infection from male (n = 4) and female (n = 4) macaques. Error bars represent standard error.

Upregulated levels of pro-inflammatory cytokines during acute stages of HIV and SIV infections have been previously reported[56]. To determine if pro-inflammatory cytokines were differentially induced based on gender, we examined the levels of plasma cytokines at day 4 and 10 PI using the 29-plex cytokine assay and compared them to day 0 values (Fig 4a–4h). Our results showed that MIG/ CXCL9, IP-10/ CXCL10, IFNγ, IL-1RA, Eotaxin, MCP-1/ CCL2, I-TAC/ CXCL11 and MIF were significantly elevated in both male and female macaques at day 10 PI as compared to day 0 and 4 PI. However, female macaques displayed significantly higher levels of plasma MCP-1 (Fig 4f), I-TAC (Fig 4g), and MIF (Fig 4h) at day 10 PI as compared to their male counterparts. All other cytokines were either below the level of detection or did not differ from day 0 values.

### Expression of IFNα subtypes- 14, 16, IFNβ and IFNω are significantly elevated in the lymph nodes of female macaques as compared to male macaques

**Fig 5:**
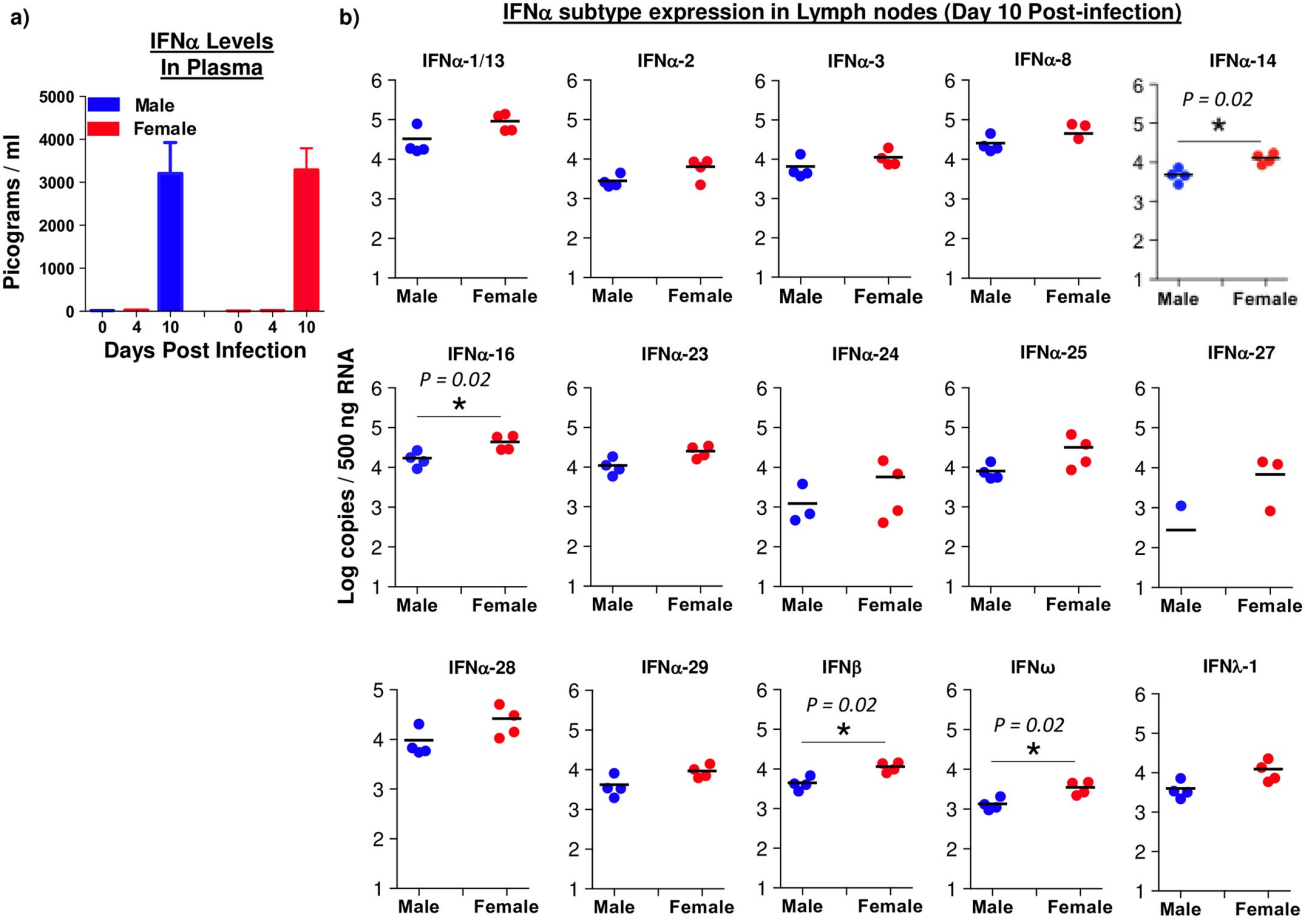
Type I (IFNα-14, 16, IFNβ, and IFNω) IFN subtypes are significantly upregulated in the lymph nodes of female macaques at day 10 post infection. (a) Levels of IFNα in the plasma samples that were collected longitudinally at day 0, 4 and 10 post-SIV infection (PI) from male (n = 4) and female (n = 4) macaques. (b) Absolute levels of Type I (IFNα-01/13, 02, 06, 08, 14, 16, 23, 24, 25, 26, 27, 28, 29, IFNβ and IFNω) and Type III (IFNλ-1) IFN transcripts in the lymph nodes of male (n = 4) and female (n = 4) macaques at day 10 PI. Exact same amount of RNA was used from each animal for quantitative analysis.

We have previously shown that plasma levels of IFNα were significantly elevated during the acute phase of SIV infection[33] whereas, others have reported significant gender based differences in induction of ISG[27], and IFNα production[57] during HIV infection To determine if gender differences in innate IFN responses were apparent *in vivo* during the acute phase of infection, we examined plasma concentrations of IFNα at day 4 and 10 PI and compared them to day 0 values. Significant levels of IFNα were detectable at day 10 PI in all the animals as compared to day 0 and 4 PI (Fig 5a) that however, did not significantly differ between male and female macaques.

Both Type I and III IFN harbor numerous subtypes that differentially regulate immune responses[33, 34]. To determine if acute SIV infection was associated with differential expression of IFN subtypes based on gender, we quantified the absolute levels of IFNα-01/13, 02, 06, 08, 14, 16, 23, 24, 25, 26, 27, 28, 29, IFNβ, IFNω and IFNλ-1 transcripts in the LN of male and female macaques at day 10 PI (Fig 5b). Our results showed that all subtypes were highly expressed in the LN of both male and female macaques. However, female macaques expressed significantly higher levels of IFNα subtype-14, 16, IFNβ and IFNω transcripts suggesting that female macaques differentially upregulate IFN responses during acute stages of SIV infection. The assay used in our study is robust and highly specific for each IFNα subtype that is coded by its own gene. Although the some of the genes are highly similar in sequence, the primers and probes were designed to detect sequences specific to each IFNα subtype. In addition, the types of probes that were used, either molecular beacons or those containing locked nucleic acids, are much more sensitive to single nucleotide differences than standard Taqman probes. In most cases, we were able to design primer-probe sets in which the probe and at least one primer was specific for its IFN-α subtype. This was verified by sequencing the PCR products using rhesus macaques. There were some subtypes for which only the probes were specific. In those cases, it is possible that non-target IFNα subtypes other than the target subtype could be amplified during the PCR reaction reducing sensitivity. Those non-target subtypes, however, are not detected due to the high specificity of the probe for each subtype suggesting that the assay used in this study could impact the sensitivity for the target gene but not the specificity of the assay for each subtype. As such we are confident that all subtypes that we detected were indeed expressed in vivo and the potential for non-specific amplification is negligible.

## Discussion

HIV infection is characterized by a progressive loss of CD4 T cells leading to adverse outcomes in therapy naïve subjects. Though the advent of anti-retroviral therapy has led to immune reconstitution and better long-term outcomes, numerous studies have documented gender differences in pathogenesis and responsiveness to therapy. Female HIV infected subjects have been shown to generate higher levels of innate and adaptive immune responses than their male counterparts; these are associated with increased immunopathogenesis[58]. Our results suggest that these differences are likely apparent during the initial stages of infection. As early as day 4 after SIV infection, CM CD4 T cells from female macaques were found to differentially regulate significantly higher number of genes and to display divergent canonical and biological pathways as compared to male macaques. By day 10 PI these differences were largely attenuated, most likely due to the significantly high level of SIV replication, though gender based differences in canonical and biological pathways were still apparent. Given the importance of CM CD4 T cells as primary targets for infection and as key players in the generation and maintenance of adaptive immune responses, these differences likely have implications for the long-term pathogenesis of HIV infection. Though our study involved only a small group of animals that needs to be validated with larger studies, gender based differences become apparent very early during the course of infection.

Earlier studies have reported upregulated levels of pro-inflammatory mediators during the early stages of HIV and SIV infections[56]. Our results were consistent with these findings as all animals showed a significant increase in plasma levels of MIG, IP-10, IFNγ, IL-1RA, Eotaxin, MCP-1, I-TAC and MIF at day 10 PI as compared the either day 0 or 4 PI. Interestingly, female macaques had significantly higher levels of MCP-1, I-TAC and MIF as compared to their male counterparts suggesting that early innate cytokine responses are skewed towards a more pro-inflammatory phenotype in female macaques.

Significantly elevated levels of MIF in the plasma of HIV infected subjects have been reported and stimulation of HIV infected PBMC with MIF was found to significantly increase HIV replication[59]. Interestingly, MIF is present in pre-formed protein in various cell types[60] and is a key regulator of inflammation[61] suggesting that inherent gender differences in the levels of pre-formed MIF likely contributed to the significantly higher levels of MIF in female macaques at day 10 PI.

Elevated levels of MCP-1, I-TAC and MIF in female macaques was surprising given the lower viral loads in HIV infected women reported in earlier studies. We did not observe a significant difference in the levels of plasma viral loads between male and female macaques that was likely due to the ramp-up phase of viremia during the 1^st^ 10 days after infection. Higher levels of pro-inflammatory mediators in the plasma of female macaques, however, suggests that acute disease is likely more severe in infected females as compared to males that may have implications for the long-term sequelae.

Previous studies have shown that higher levels of I-TAC, MCP-1 etc. was associated with increased immune activation[62]. High levels of MCP-1, MIP-1α and β, IL-4, IL-10 and RANTES expression was shown to correlate with pathogenic outcomes during primary SIV infection[63]. Unlike MCP-1, I-TAC is a chemotactic chemokine that preferentially recruits CXCR3+ T cells that have been implicated in cardiovascular diseases such as atherosclerosis[64–66]. Additional longitudinal studies are needed to better understand the implications of these differences on long-term pathogenic consequences of infection.

Others have reported sex-based differences in the production of IFNα[28, 57]. We did not observe a significant difference in plasma levels of IFNα in male and female macaques likely due to acute viral replication. However, a number of IFN subtypes (IFNα-14, 16, IFNβ, and IFNω) were significantly elevated in the LN at day 10 PI suggesting that there were gender based differences in the early innate IFN response to SIV infection. We were unable to get samples for healthy male and female macaques and cannot completely rule out if there were any inherent differences in IFN subtype responses in male vs female healthy animals. Studies have shown that pDC were actively recruited from circulation into the LN and were the primary producers of Type I IFNα during acute SIV infection[34]. Others have shown that pDC produce up to 1000-fold more IFN-α/β and IFN-λ than other cell types[67]. Zeigler et al[68] reported that pDC’s from female subjects when stimulated with TLR7 agonists induced significantly higher levels of IFNα subtypes and IFNβ as compared to pDC from male subjects. Others have reported that sex differences in IRF5 levels in pDC contribute to the induction of high levels of IFNα in female subjects as compared to males[69]. The exact reasons why these subtypes were significantly higher in female macaques as compared to male macaques are not clear. It is possible that the gender differences in pattern recognition receptors such as TLR7 and TLR8 that have been shown to play a major role in the production of IFNα during SIV infection contributes to this process. Both TLR7 and TLR8 are expressed on the X-chromosome[70–72] and TLR7 mediated induction of higher levels of IFNα in females have been previously reported[73]. On the other hand, it is also possible that gender differences in inflammatory responses likely contribute to the differential induction of IFN responses. Meier et al[28] reported that higher immune activation in HIV infected female patients was associated with sex differences in TLR mediated responses of pDC.

The significance of differential induction of IFN subtypes in the face of massive viral replication at day 10 PI is not clear though induction of higher IFNα responses have been associated with increased immune activation during HIV infection[74–76]. Likewise, higher levels of IFNα production was associated with increased T cell activation in treatment naïve HIV infected women as compared to men when adjusted for viral loads[28]. It is possible that early IFN responses in combination with other cytokines, likely play a role in shaping the course of immune activation during HIV infection. Abel et al reported that IFN-α/β responses were induced during the acute phase of SIV infection[77], whereas elevated levels of IFNα have been reported in the sera of HIV-1-infected and AIDS patients[78]. Sooty mangabeys, the natural hosts for SIV infection express less ISG during chronic infection as compared to pathogenic hosts and display substantially reduced levels of immune activation during SIV infection[79]. Other studies have reported that natural hosts produce high levels of IFNα during acute stages of infection but these responses are more rapidly resolved[79, 80]. Interestingly, IFNα-14 has been shown to induce high levels of protective ISG such as MX2 and Tetherin and reduce viral replication[81–83]. Treatment of HIV infected NSG (NOD-scid IL-2rγc^null^) mice transplanted with human peripheral blood mononuclear cells or humanized mice with IFNα-14 and IFNβ significantly decreased HIV replication *in vivo* as compared to other subtypes such as IFNα-2, IFNα-6 and IFNα-8[82, 84]. Whether induction of these IFN subtypes alters the course of HIV pathogenesis in female subjects as compared to males is still not clear and needs to be examined in future studies.

In conclusion, our studies support earlier studies showing gender based differences in driving HIV pathogenesis and suggest that pathogenic consequences observed during the chronic stages of disease are likely shaped very early during the course of infection. Additional larger studies are needed to better characterize the effect of gender during the early acute phases of infection, and the potential long-term consequence of these differences on disease progression and associated co-morbidities.

## Supporting information

S1 Fig Female macaques differentially regulate significantly larger number of genes during acute SIV infection as compared to male macaques—Individual animal data. Heat map generated using genes that were differentially regulated in CM CD4 T cells from male and female macaques at day 4 and 10 post SIV infection. Each animal’s day 0 sorted CM CD4 T cell sample was used to compare to the same animal’s day 4 and 10 post SIV infection sorted samples. The same genes as in Fig 2C are depicted and the expression profiles for each of the genes is visualized for each individual animal in the study.
(TIF)

S2 Fig Housekeeping genes GAPDH and 18s amplified at the same Ct in all the animals. Relative levels of GAPDH and 18s in lymph node mRNA samples from male (n = 4) and female (n = 4) macaques that were used for quantifying Type I IFN subtype responses.
(TIF)

S1 Table List of genes from all the male (n = 4) and female (n = 4) macaques that showed a minimum of two-fold change in expression (up or down) at day 4 and day 10 as compared to day 0. (XLSX)

S2 Table Genes whose expression is differentially regulated in male versus female macaques at 4 days post infection. (XLSX)

S3 Table Genes whose expression is differentially regulated in male versus female macaques at 10 days post infection. (XLSX)
