# Supplementary figures and images for: Gender differences in innate responses and gene expression profiles in memory CD4 T cells are apparent very early during acute simian immunodeficiency virus infection
Source: PLoS One. 2019 Sep 6;14(9):e0221159. doi: 10.1371/journal.pone.0221159 (PMC6730907; doi:10.1371/journal.pone.0221159)

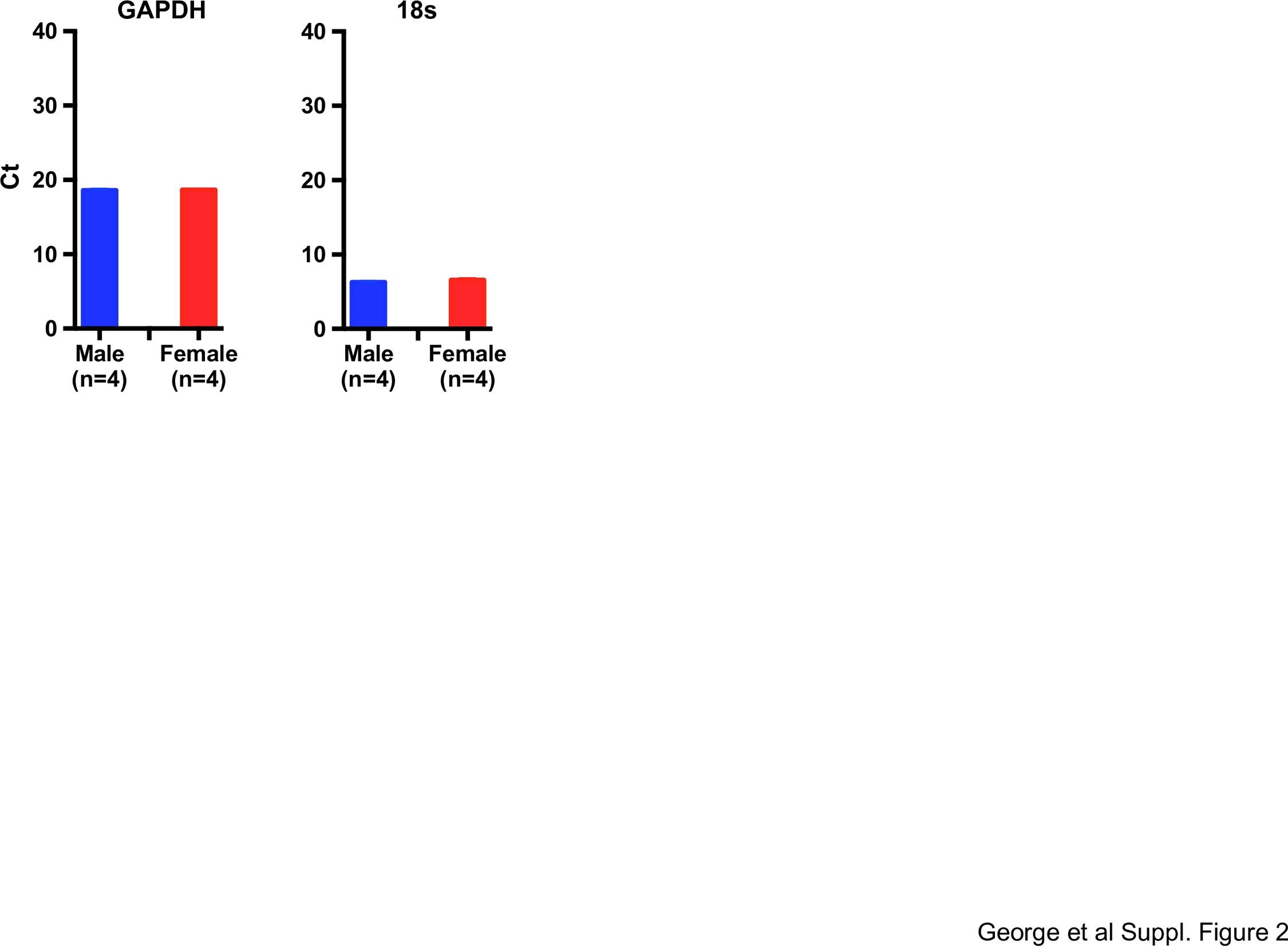

Supplement: S1 Fig — Heat map generated using genes that were differentially regulated in CM CD4 T cells from male and female macaques at day 4 and 10 post SIV infection. Each animal’s day 0 sorted CM CD4 T cell sample was used to compare to the same animal’s day 4 and 10 post SIV infection sorted samples. The same genes as in Fig 2C are depicted and the expression profiles for each of the genes is visualized for each individual animal in the study. (TIF) [file pone.0221159.s001.tif]

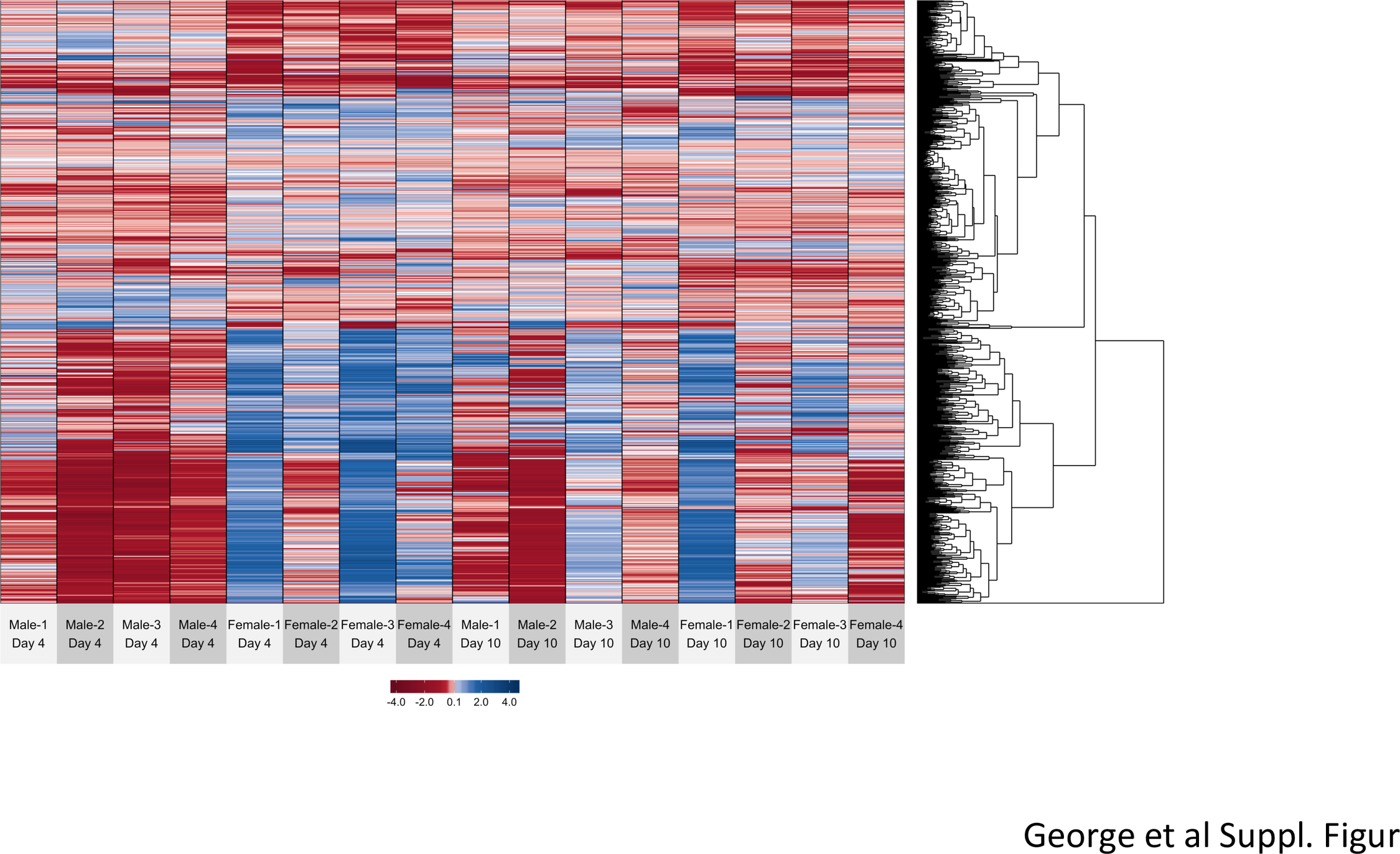

Supplement: S2 Fig — Relative levels of GAPDH and 18s in lymph node mRNA samples from male (n = 4) and female (n = 4) macaques that were used for quantifying Type I IFN subtype responses. (TIF) [file pone.0221159.s002.tif]
